# Preclinical and clinical evaluation of the Janus Kinase inhibitor ruxolitinib in multiple myeloma

**DOI:** 10.18632/oncotarget.28547

**Published:** 2024-02-05

**Authors:** Ashley Del Dosso, Elizabeth Tadevosyan, James R. Berenson

**Affiliations:** ^1^ONCOtherapeutics, West Hollywood, CA 90069, USA; ^2^Berenson Cancer Center, West Hollywood, CA 90069, USA; ^3^Institute for Myeloma and Bone Cancer Research, West Hollywood, CA 90069, USA; ^*^These authors contributed equally to this work

**Keywords:** multiple myeloma, ruxolitinib, JAK/STAT, cytokine, clinical trial

## Abstract

Multiple myeloma (MM) is the most common primary malignancy of the bone marrow. No established curative treatment is currently available for patients diagnosed with MM. In recent years, new and more effective drugs have become available for the treatment of this B-cell malignancy. These new drugs have often been evaluated together and in combination with older agents. However, even these novel combinations eventually become ineffective; and, thus, novel therapeutic approaches are necessary to help overcome resistance to these treatments. Recently, the Janus Kinase (JAK) family of tyrosine kinases, specifically JAK1 and JAK2, has been shown to have a role in the pathogenesis of MM. Preclinical studies have demonstrated a role for JAK signaling in direct and indirect growth of MM and downregulation of anti-tumor immune responses in these patients. Also, inhibition of JAK proteins enhances the anti-MM effects of other drugs used to treat MM. These findings have been confirmed in clinical studies which have further demonstrated the safety and efficacy of JAK inhibition as a means to overcome resistance to currently available anti-MM therapies. Additional studies will provide further support for this promising new therapeutic approach for treating patients with MM.

## INTRODUCTION

### Pathophysiology of multiple myeloma

Multiple myeloma (MM) is a blood cancer of monoclonal plasma cells that accumulate in the bone marrow (BM) and produce M-protein (also known as monoclonal immunoglobulin or paraprotein). MM is the second most prevalent hematological malignancy, accounting for nearly 2% of cancer diagnoses and related deaths in the United States [1]. It exhibits a notable demographic bias, with a higher incidence among the elderly, men, and African Americans [1]. Within the spectrum of disorders known as monoclonal gammopathies, MM finds its place alongside monoclonal gammopathy of unknown significance (MGUS) and smoldering myeloma (SM).

Patients typically present with symptoms related to organ dysfunction: hypercalcemia, renal insufficiency, anemia, and bone destruction (known as the CRAB criteria). While MM remains incurable, recent therapeutic advancements have increased the average five-year survival rate from 25% in 1975 to 40% in 2008 [2, 3]. A recent study of unselected MM patients from our clinic shows a median survival of 136.2 months [4]. Although the etiology of MM remains elusive, decades of research have established a foundational understanding of the mechanisms of drug resistance [5, 6].

Understanding MM as a malignancy rooted in terminally differentiated plasma cells necessitates a comprehensive grasp of the BM microenvironment, terminal B-cell biology, and immunology which are all pivotal players in identifying potential treatment avenues. Within the BM reside hematopoietic stem cells, capable of differentiating into various blood cell types while sustaining their own population through self-renewal. Among their progeny, B cells hold significance, as they have the capability to produce a wide variety of antibodies, alerting the adaptive immune system to biological threats and eventually giving rise to antibody-producing plasma cells [7].

While numerous factors contributing to malignancy are intrinsic to tumor cells, they often lack the capability to independently drive progression and metastasis. The emergence of full-blown malignancy, along with the subsequent evasion of apoptosis, typically necessitates a supportive or even favorable microenvironment. Beyond B cell-related mutations, the BM microenvironment, consisting of mesenchymal stromal cells, fibroblasts, adipocytes, endothelial cells, osteoclasts, osteoblasts, immune cells, and hematopoietic cells, plays a crucial role in MM progression. This complex population has the capability to induce myeloma cell migration to the BM and the erratic proliferation of myeloma cells while also mediating survival, proliferation, drug resistance, and progression of the disease [8, 9]. The intricate interplay within the BM microenvironment, coupled with the diverse subclonal makeup of MM cell populations [10], significantly contributes to the resistance seen in MM patients to their treatment regimens [8, 9]. Consequently, despite significant progress in understanding MM and the availability of a wide range of therapeutic modalities, including immunomodulatory agents (IMiDs), proteasome inhibitors (PIs), monoclonal antibodies, histone deacetylase inhibitors, selective inhibitors of nuclear export, bispecific antibodies, and CAR-T cell therapies, MM remains an incurable disease. This current inability to effectively treat patients long term highlights the need for therapeutic options capable of augmenting and extending the efficacy of currently available treatments.

### JAK/STAT signaling in cancer - inhibition as a potential treatment

The JAK/signal transducer and activator of transcription (STAT) pathway is a membrane-to-nucleus signaling module responsible for instigating the expression of numerous regulators associated with both cancer and inflammation. This pathway encompasses an extensive array of more than 50 cytokines and growth factors, including hormones, interferons (IFN), interleukins (ILs), and colony-stimulating factors [11]. Consequently, JAK/STAT-mediated downstream processes exhibit a diverse range of critical functions including hematopoiesis, immune competence, tissue regeneration, inflammatory responses, and regulation of apoptosis [12].

Under normal conditions, JAK and its downstream transcription factors, STAT proteins, mediate hematopoietic cytokine receptor signaling [13, 14] which in turn affects cell growth, survival, and differentiation through many cellular events [13, 14]. Within the context of myeloproliferative neoplasms, it has been reported that through mutations, JAK proteins can become constitutively active and consequently promote the survival and proliferation of abnormal cells, a process thought to contribute to the development of lymphoma, leukemia, and more recently MM [8, 13, 14]. Additionally, elevated levels of growth factors and cytokines in MM have been shown to contribute to increased JAK2 activation [8]. IL-6, a growth and survival factor for myeloma cells, is among those cytokines that activate JAK2 and ultimately augment its downstream signaling effects [8]; and, therefore, JAK inhibitors represent potential therapies for treating MM patients.

Ruxolitinib (RUX), an oral JAK inhibitor approved by the FDA for the treatment of myelofibrosis, polycythemia vera, and graft versus host disease works through reducing the abundance of cytokines and growth factor receptors that utilize JAK1 and JAK2. MM implicates JAK1 and JAK2 genes in its pathogenesis, much like myelofibrosis does [15]. Myelofibrosis is a clonal disorder originating at the level of the hematopoietic stem cell and is characterized by BM fibrosis, splenomegaly, and extramedullary hematopoiesis [15, 16]. In the randomized, phase 3 COMFORT-II trial (Controlled Myelofibrosis Study with Oral Janus-Associated Kinase Inhibitor Treatment-II), RUX treatment showed superiority to the best currently available therapy at the time [17]. Specifically, it was found to rapidly reduce splenomegaly and other debilitating symptoms of myelofibrosis [17]. These results demonstrate the beneficial effects of RUX on quality of life compared with the current best available therapies in myelofibrosis. In contrast to RUX, the best available therapy was associated with an increase in spleen volume and a worsening of symptoms [17]. The success of JAK inhibitors in myelofibrosis has prompted preclinical experiments in other hematologic cancers, specifically MM, owing to similarities in their pathogenesis. Success in preclinical research using JAK inhibitors for the treatment of MM has further prompted early-phase clinical studies. For example, RUX is being studied as part of an all-oral treatment regimen [18–20] that addresses many of the current issues that occur with regimens containing IMiDs, PIs, and monoclonal antibodies. The following sections of this article will be focused on studies of RUX in the preclinical [21–24] and clinical settings [18–20] focused on the treatment of relapsed/refractory (RR) MM.

### Preclinical evaluation of ruxolitinib in multiple myeloma

Checkpoint proteins play a critical role in T-cell regulated immune responses and self-tolerance. Expression of the key checkpoint protein, programmed cell death ligand-1 (PD-L1), is partly regulated by the IL-6/STAT3 signaling pathway. Its overexpression in MM is likely related to the upregulation of IL-6 levels often seen in the disease and, consequently, enhanced downstream JAK/STAT signaling [25–27]. Additionally, it has been shown that while PD-L1 expression can be detected on plasma cells from MM patients, it is rarely detected on plasma cells from healthy donors [28, 29]. This increased expression occurs not only on the surface of malignant cells but also other cell types in the BM stroma [30, 31]. This leads to the development of exhausted T cells within the MM microenvironment, impairing the function of cytotoxic T cells and facilitating tumoral escape and resistance to immune-based approaches for treating MM patients.

Immune checkpoint inhibitors that target cytotoxic T lymphocyte antigen 4 (CTLA-4), PD-1 and PD-L1 have been shown to enhance the efficacy of many cancer therapies [32, 33]. However, anti-PD-1/PD-L1 therapy is only effective in a minority of patients with melanoma, non-small cell lung and renal cell cancers [34]. Clinical trials involving checkpoint inhibitors alone have lacked consistent efficacy and have yielded significant safety concerns when used to treat MM patients [35–37]. In a recent preclinical study, we have shown that treatment with RUX is capable of increasing MM cell apoptosis when combined with IL-2-stimulated T cells [23]. This is similar to the effects of anti-PD-1 or anti-PD-L1 antibodies but does not appear to be associated with the immune-related adverse events (IRAEs) that commonly occur with the administration of these types of antibodies [18, 19, 25]. We have shown that treatment of MM patients’ BM mononuclear cells (MCs) with RUX, even at low concentrations, reduces both the proportion of PD-L1 expressing cells and levels of PD-L1 expression [23]. Additionally, it was shown that RUX was able to reduce PD-L1 expression in HS-5 stromal cells and increase the cytotoxic effects of T cells on MM tumor cells in a co-culture of IL-2 stimulated T-cells with MM BMMCs. Comparable data was later collected by our group regarding another immune checkpoint protein, B7-H3 [24]. Treatment of primary BMMCs from MM patients with progressive disease (PD) and three MM cell lines (RPMI8226, U2666 and MM1S) with RUX reduced B7-H3 expression. Additionally, in both MM BMMCs cultured alone or in combination with SUP-T1 T-cells, treatment with RUX resulted in increased IL-2 expression and CD8 expression, further supporting that inhibition of the JAK/STAT signaling pathway through RUX is capable of reducing the expression of checkpoint proteins; and, therefore, increases the cytotoxic capacity of T-cells against MM tumor cells, potentially limiting tumoral escape and resistance to immune based approaches in MM treatment.

Among the key players in the MM microenvironment are macrophages. Depending on the signals they receive from support cells such as BM stromal cells, they can take on a pro-inflammatory (M1) or an alternative (M2) immune inhibitory phenotype [38]. M1 macrophages possess anti-tumor effects and are decreased in the BM of MM patients with PD while M2 macrophages are increased [22, 39] and have been shown to promote tumor growth and metastasis through the secretion of growth factors and associated promotion of angiogenesis [40–42]. Tribbles homolog 1 (TRIB1) is a myeloid oncogene and a potent inducer of M2 polarization [43, 44]. The expression of TRIB1 along with the transmembrane glycoprotein MUC1, the MM cell surface adhesion receptor CD44, and the chemokines CXCL12 and CXCR4 are additionally upregulated in the BM of MM patients [45, 46]. The increased expression of these factors is the result of complex, context dependent, feedback loops involving the inflammatory tumor microenvironment, immune cells, and myeloma cells. An integral signaling pathway implicated in MM is the JAK/STAT pathway which has been shown to directly activate the MAPK and mTOR pathways, additionally influencing cell proliferation, differentiation, and survival [11, 12, 14]. The JAK/STAT pathway is also capable of indirectly influencing the WNT/β-catenin pathway which plays a role in regulating the balance of osteoclasts and osteoblasts, as well as in the regulation of MUC1 expression [47, 48].

We have recently shown both *in vitro* and *in vivo* that RUX is capable of blocking M2 macrophage polarization through several mechanisms [22]. Treatment of a co-culture consisting of MM BMMCs from patients with PD and normal peripheral blood (PB) MCs showed a significant increase in the polarization of macrophages to the M2 state after one week in culture. Notably, RUX reduced expression of the M2 marker CD36 and expression of the MAPK/ERK pathway regulator TRIB1 in the human leukemia monocytic line (THP-1) when cultured with MM cell lines. Similarly, RUX treatment of human MM xenograft LAGκ-1A tumors growing in severe combined immune deficient mice resulted in reduction of M2 CD36 expression and an increase in expression of the M1 marker CD86 [22]. We also found increased levels of CXCL12 in BM plasma from MM patients and in the supernatants from cultured MM BM stromal cells. Therefore, these results show that MM tumor cells can drive M2 macrophage polarization. Consequently, as a response to increased cytokines in the MM BM microenvironment, signaling molecules involved in the JAK/STAT pathway and WNT signaling pathway may mediate M2 polarization by increasing TRIB1 and CXCL12/CXCR4 expression in monocytes. Activation of the CXCL12/CXCR4 axis upregulates TRIB1 gene expression through HOXA9/MEIS1 expression [49]. The CXCL12/CXCR4 axis, which we have shown to be overactive in MM BM, has been shown to also upregulate MUC1 and CD44 gene expression [48, 50]. M2 macrophages induced by MM in turn upregulate both MUC1 and CD44 on tumor cells [22]. Upregulation of MUC1 and CD44 has been shown to be associated with resistance to the highly used IMiD, lenalidomide (LEN), in MM [48] and the ability of RUX to downregulate their expression, potentially serving as a means to overcome resistance, is an attractive benefit of this JAK inhibitor. Recent studies also show that M2 macrophages have markedly increased expression of PD-L1 when compared to M1 macrophages [51, 52]. The increase in M2 macrophages and accompanying PD-L1 expression likely contributes to the high frequency of drug resistance seen in MM. This limits the ability of T cells to target malignant cells for killing which is reversed by RUX.

Some insight into the ability of JAK inhibitors to perform synergistically with currently available MM treatments, including IMiDs, has recently been gained by our group through *in vitro* and *in vivo* analyses of the preclinical anti-MM effects of another selective JAK1 inhibitor INCB052793 [21]. In the MM cell line RPMI8226, the proportion of apoptotic and necrotic myeloma tumor cells among cells treated with INCB052793 in combination with the PIs carfilzomib or bortezomib was markedly higher than cells exposed to single agents. Similarly, using fresh tumor cells from MM patients, this same doublet combination produced a higher percentage of total cell death (early and late apoptosis and necrosis) than exposure of these tumor cells to single agents. This effect remained consistent across drug classes including the immunomodulatory agent LEN and the glucocorticosteroid DEX. RUX was able to reduce STAT3 activation and increase apoptosis of myeloma cells in both IL-6 independent and IL-6 dependent lines.

These preclinical findings regarding the anti-myeloma effects of RUX and other JAK inhibitors suggest that these drugs may be a promising addition to the current standard of therapy used to treat MM patients and warrants further clinical assessment of this JAK inhibitor in this patient population.

### Clinical evaluation of JAK inhibitors in multiple myeloma

LEN, an IMiD, exerts its diverse anti-cancer effects within both cancer cells and the surrounding microenvironment. This includes inducing alterations in cytokine production, immune cell activity, and, in some cases, inflammation and the death of tumor cells [53]. However, over time, patients with MM acquire resistance to this drug, underscoring the need for therapeutic strategies to address drug resistance.

It is believed that RUX’s ability to overcome LEN resistance results from multiple factors, which includes its effects on the WNT/B-catenin/CD44 pathway and the inhibition of MUC1 [22, 48]. Resistance to LEN is linked to the upregulation of key factors such as MUC1, β-catenin, MYC, and CD44. Notably, MUC1 serves to stabilize β-catenin, forming a complex that targets specific WNT-responsive genes like MYC and CCND1, thereby enhancing gene transcription, as evidenced by heightened CD44 expression [22, 48]. Indeed, the analysis of MM cell lines has unveiled a correlation between increased CD44 levels and LEN resistance. The inhibition of MUC1 leads to enhanced proteasomal degradation of β-catenin, which, in turn, reduces WNT gene transcription and results in decreased CD44 expression [48, 54]. Furthermore, this disruption in redox balance triggers heightened apoptosis via reactive oxygen species, ultimately reinstating sensitivity to LEN.

Based on these findings, a phase 1 study was conducted to assess the efficacy of RUX, LEN, and steroids for patients with RRMM [19]. Twenty-eight patients participated in the initial part of the clinical trial; they had received a median of 6 prior treatments (range, 3–14) including LEN and steroids, to which 93% were refractory. In the first part of this study, participants were divided into four cohorts using a standard 3+3 dose escalation design. All subjects received an entirely oral treatment regimen consisting of a 28-day cycle of RUX, twice daily from days 1 to 28, LEN, once daily from days 1 to 21, and the glucocorticosteroid methylprednisolone every other day from days 1 to 28. No dose-limiting toxicities were observed at the maximal administered dose (MAD) consisting of 15 mg RUX, 10 mg LEN, and 40 mg methylprednisolone. Of the 28 patients, 26 were refractory to their last LEN treatment and 69% (*n* = 18) of these patients were treated with ≥10 mg of LEN with an overall response rate (ORR) of 29% and a 41% clinical benefit rate (CBR). Notably, 72% (*N* = 13) of the 18 patients had undergone treatment with a LEN-containing regimen immediately prior to joining this study. Among these 13 patients, the responses included one complete response (CR), one very good partial response (VGPR), three partial responses (PR), one minimal response (MR), and seven cases of stable disease (SD). Overall, the CBR and ORR observed in this study was 46% and 38%, respectively and notably, all 12 patients who responded were refractory to LEN. Given the promising findings and exceptional tolerability demonstrated in this study, the trial was expanded to encompass an additional cohort of 21 patients, thereby increasing the total study population to 49 patients. Newly enrolled patients were treated at the MAD, consisting of RUX 15 mg, LEN 10 mg, and methylprednisolone 40 mg. These 21 patients and those treated as part of the dose escalation phase of the study (*N* = 28) were recently reported together (*N* = 49) [20]. Patients had received a median of 6 prior treatments (range, 3–15), including LEN and steroid-containing regimens, of which 94% were refractory. The CBR was 49% and the ORR was 36%. The median progression free survival (PFS) for all evaluated patients was 3.5 months, and the duration of response (DOR) for 22 patients who were observed to have MR or better was 7.2 months. Overall, the treatment was well tolerated, and the observed adverse events were consistent with established toxicities associated with the respective medications. We have recently reported that baseline serum B-cell maturation antigen (sBCMA) levels predicted outcomes for RRMM patients starting new therapies [55]. Similarly, we found that lower baseline sBCMA levels were associated with a longer PFS in this study [56, 57].

Furthermore, our preclinical studies suggested that JAK inhibitors may show clinically significant effects even in the absence of immunomodulatory agents to treat MM patients [21–24]. RUX’s ability to reverse desensitization to dexamethasone in MM patients involves a complex interplay between the JAK/STAT pathway and glucocorticosteroids. Notably, the administration of dexamethasone has been observed to elevate both STAT3 and the pro-survival factor PI3K levels within melanoma cells, consequently leading to an increase in STAT3 levels [58]. Thus, prolonged exposure to dexamethasone often leads to the development of resistance. However, this resistance can be partially overcome by inhibiting the JAK/STAT pathway [58, 59], highlighting the potential of JAK inhibitors like RUX to restore sensitivity to dexamethasone treatment, as well.

Considering these observations, the phase 1 study was amended to assess the efficacy of RUX with steroids alone for treating RRMM patients [18]. The study enrolled and treated 29 patients who had previously undergone extensive prior treatments, including a median of 6 (range 3–12) lines of therapy, all with prior exposure to PIs and LEN. The treatment regimen involved the administration of RUX at a dose of 15 mg twice daily, along with 40 mg of oral methylprednisolone every other day. Notably, 9 patients exhibited at least a partial response (PR) to the treatment, with one patient achieving a very good (VG) PR and 8 attaining PRs. This translated to an ORR and CBR of 31%. The DOR was encouraging at 13.1 months. Furthermore, those who responded to the therapy displayed a median PFS of 15.6 months, while non responders showed a significantly shorter PFS of 1.6 months. This was the first clinical study to demonstrate the efficacy of JAK inhibitors combined with steroids alone for the treatment of MM patients. Similar to what was observed with the addition of LEN to this combination, higher baseline sBCMA levels correlated with a shorter PFS (JRB, manuscript in preparation), offering valuable insights and encouraging further investigation into potential predictive factors for this regimen for treating MM patients. As part of the study, 20 patients who were failing the two-drug combination had LEN at 10 mg daily added to the regimen and showed an ORR of 30% and CBR of 40%. Notably, 71% of patients who had initially achieved a MR or better responded to the addition of LEN, whereas only 23% of patients who did not achieve better than a MR responded to the addition of this IMiD [60]. Overall, responses following the addition of LEN were as follows: 2 VGPR, 4 PR, 2 MR, 8 SD, and 4 PD. The median PFS was 3.5 months and the PFS2 (time from start of first treatment to end of the study) was 7.3 months. Interestingly, as noted with the previously discussed studies, sBCMA levels were predictive of PFS2 with those showing lower levels showing a longer survival.

Because of these encouraging findings, the current trial (clinical trials.gov number, NCT03110822) has been expanded to include treatment of the RUX/LEN/methylprednisolone at the MAD for those with impaired renal function, a common occurrence among patients with RRMM [61]. In addition, since RUX is used at higher doses to treat those with myeloproliferative disorders, the current study has also added an arm that includes a 20 mg dose of RUX BID with methylprednisolone.

Considering the favorable outcomes observed with RUX, subsequent studies have been initiated to evaluate the efficacy of alternative JAK inhibitors in the management of MM, as well as other hematologic malignancies. Phase 1 of the phase 1/2 study was conducted to assess INCB052793, a potent JAK1 inhibitor, alone and in combination with standard therapies for patients with MM, acute myeloid leukemia (AML), and myelodysplastic syndrome (MDS) [62]. The Phase 2 study examined the combination of itacitinib with azacitidine among patients with AML and MDS, and did not pertain to MM. The study utilized a phase 1a/1b design to assess INCB052793’s safety, tolerability, and efficacy. Phase 1a involved a dose escalation using INCB052793 as a monotherapy in patients with MM, refractory to at least two prior therapies, following a 3+3 design. Phase 1b evaluated INCB052793 in combination with dexamethasone in MM patients. Monotherapy in Phase 1a of the study showed that all MM patients experienced treatment-emergent adverse events of grade 1 or higher. Unfortunately, no therapeutic responses were observed in this cohort. In phase 1b, 2 out of 7 MM patients exhibited minimal response when treated with INCB052793 and dexamethasone, indicating limited efficacy. The study was terminated prematurely due to a lack of efficacy across all patient populations and therapy combinations. Despite promising preclinical data suggesting the potential of INCB052793 and itacitinib, this clinical trial faced significant challenges in achieving therapeutic responses in MM patients.

## DISCUSSION

The JAK family, which includes JAK1 and JAK2, plays a pivotal role in the pathogenesis of MM [14, 63, 64]. Within the BM microenvironment of MM patients, cytokines have been observed to trigger the activation of the JAK/STAT signaling pathways within tumor cells. This activation, in turn, contributes significantly to the growth, survival, and development of drug and resistance that occurs in MM patients. The preclinical and clinical studies included in this article highlight the potential of the JAK inhibitor, RUX, to treat heavily, previously treated RRMM patients in combination with steroids as well as LEN, even among those resistant to both drugs. Its capacity to overcome resistance mechanisms and synergistically complement existing treatments holds promise for enhancing the therapeutic strategies employed against this incurable disease. Importantly, RUX is well tolerated and associated with few side effects. Additionally, its oral administration provides a convenient treatment option for patients which also holds true when combined with steroids and LEN.

**Figure 1:**
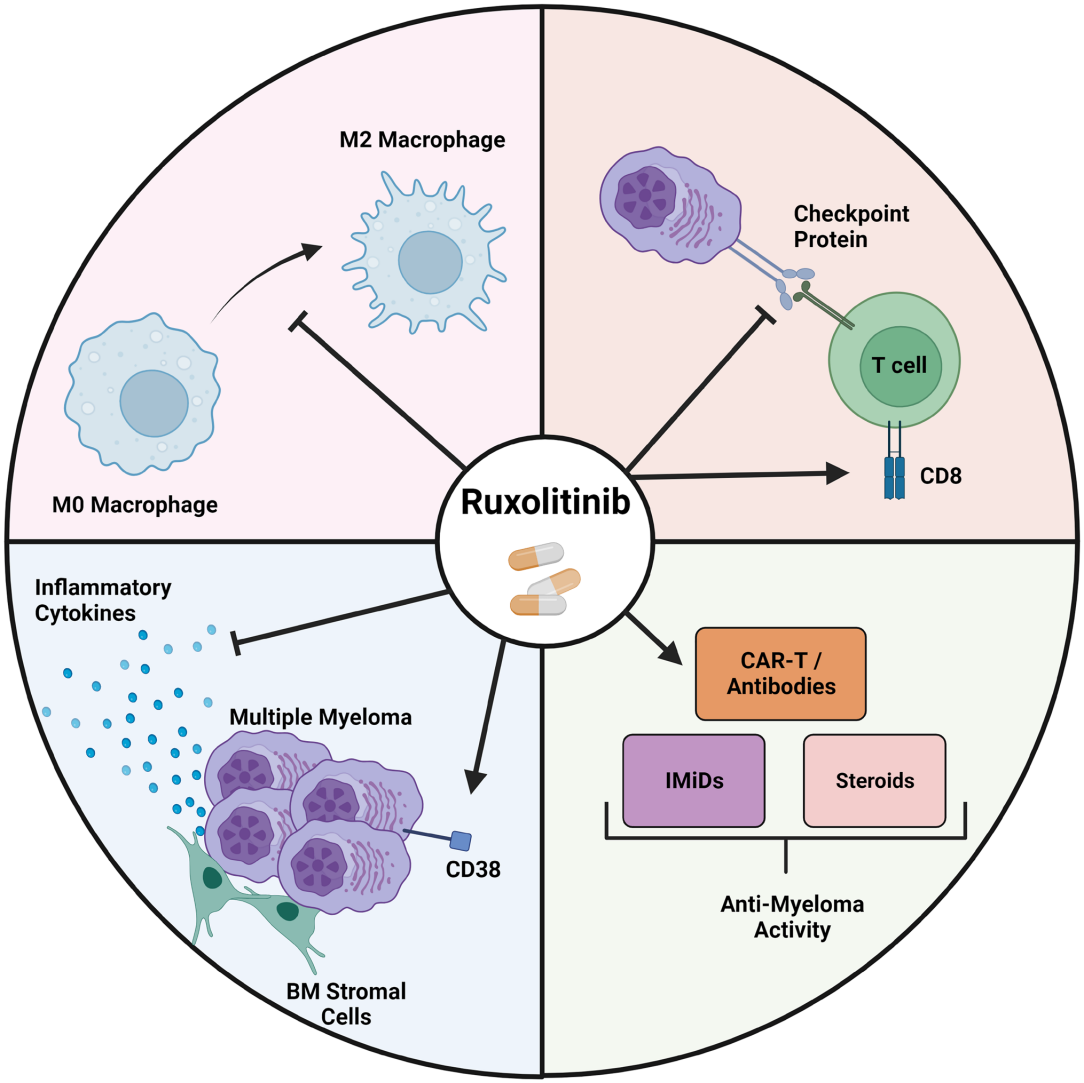
Ruxolitinib exerts its anti-myeloma effects through several mechanisms. Ruxolitinib is able to block M2 polarization in macrophages (top left) and is able to inhibit the expression of immune checkpoint proteins while at the same time increasing the cytotoxic activity of T-cells (top right). Additionally, through direct inhibition of the JAK/STAT pathway and indirect interactions with the WNT/β-catenin pathway, RUX is able to reduce the production of inflammatory cytokines from myeloma cells and BM stromal cells while increasing CD38 expression on myeloma cells (bottom left) which augments the activity of established anti myeloma agents (bottom right).

Preclinical studies and clinical studies have suggested other areas of potential usefulness for JAK inhibitors for treating MM patients (Figure 1) based on its ability to increase the expression of CD38 on MM tumor cells and reduce the risk and severity of IRAEs from bispecific antibody and CAR-T cell therapies while simultaneously augmenting the anti-tumor effects of these immune-based therapies. First, RUX increases the expression of CD38 on MM cells [65], and as a result, this may increase the efficacy of the commonly used anti-CD38 antibodies daratumumab and isatuximab to treat MM patients [66, 67]. Second, despite the fact that RUX impairs vaccination responses to COVID-19 among patients with MM and myeloproliferative disorders [68–70], RUX and other JAK inhibitors have been shown to improve outcomes for treating hospitalized patients with COVID-19 infection [71]. In addition, abrupt withdrawal of RUX among patients infected with COVID-19 has been shown to lead to poor outcomes [72]. This effect is thought to be related to the ability of RUX to shutdown inflammatory responses brought on by high levels of inflammatory cytokines associated with COVID-19 infection [73]. Thus, it is possible that treatment of patients undergoing CAR T-cell or bispecific antibody therapy with RUX may mitigate cytokine release syndrome and neurologic side effects that commonly impact patients undergoing these newer and highly effective anti-MM therapies [74, 75]. Third, given the inhibitory effects of RUX on checkpoint inhibitor gene and protein expression as we have demonstrated in MM and others have shown in other malignancies combined with its ability to augment anti-MM effects of T-cells [22–24], it is possible that RUX may improve the efficacy of immune-based therapies that rely on T-cell activity including CAR T-cell and bispecific antibody treatments. Thus, the results of the studies presented in this review will hopefully provide the impetus for conducting additional preclinical and clinical studies to evaluate RUX in the setting of MM as well as other types of cancer.

## Abbreviations

MM: Multiple Myeloma
JAK: Janus Kinase
SM: Smoldering Myeloma
MGUS: Monoclonal Gammopathy of Undefined Significance
HSC: Hematopoietic Stem Cell
MSC: Mesenchymal Stromal Cell
EC: Endothelial Cell
OC: Osteoclast
OB: Osteoblast
IMiD: Immunomodulatory Drug
PI: Proteasome Inhibitor
IFN: Interferon
IL: Interleukin
RR: Relapsed/Refractory
IRAEs: Immune-Related Adverse Events
BMMC: Bone Marrow Mononuclear Cell
AE: Adverse Event
SEA: Severe Adverse Event
PBMC: Peripheral Blood Mononuclear Cell
SCID: Severe Combined Immunodeficiency
PD: Progressive Disease
CR: Complete Response
PR: Partial Response
MR: Minimal Response
SD: Stable Disease
VGPR: Very Good Partial Response
MAD: Maximum Administered Dose
PFS: Progression Free Survival
DOR: Duration of Response
sBCMA: Serum B Cell Maturation Antigen
CBR: Clinical Benefit Rate
ORR: Overall Response Rate
AML: Acute Myeloid Leukemia
MDS: Myelodysplastic Syndrome
CAR-T: Chimeric Antigen Receptor – T Cell
